# Cancer Nano-Immunotherapy from the Injection to the Target: The Role of Protein Corona

**DOI:** 10.3390/ijms21020519

**Published:** 2020-01-14

**Authors:** Idoia Mikelez-Alonso, Antonio Aires, Aitziber L. Cortajarena

**Affiliations:** 1 CIC biomaGUNE, Parque Científico y Tecnológico de Gipuzkoa. Paseo de Miramón 182, 20014 Donostia-San Sebastián, Spain; imikelez@cicbiomagune.es (I.M.-A.); aaires@cicbiomagune.es (A.A.); 2 Immunopathology, BiocrucesBizkaia, Cruces Plaza, 48903 Barakaldo, Spain; 3 Ikerbasque, Basque Foundation for Science, 48013 Bilbao, Spain

**Keywords:** cancer immunotherapy, nanoparticles, protein corona, personalized protein corona, nanomedicine, immune response, immune blinding, immune reactivity, checkpoint inhibitors, personalized medicine

## Abstract

Immunotherapy has become a promising cancer therapy, improving the prognosis of patients with many different types of cancer and offering the possibility for long-term cancer remission. Nevertheless, some patients do not respond to these treatments and immunotherapy has shown some limitations, such as immune system resistance or limited bioavailability of the drug. Therefore, new strategies that include the use of nanoparticles (NPs) are emerging to enhance the efficacy of immunotherapies. NPs present very different pharmacokinetic and pharmacodynamic properties compared with free drugs and enable the use of lower doses of immune-stimulating molecules, minimizing their side effects. However, NPs face issues concerning stability in physiological conditions, protein corona (PC) formation, and accumulation in the target tissue. PC formation changes the physicochemical and biological properties of the NPs and in consequence their therapeutic effect. This review summarizes the recent advances in the study of the effects of PC formation in NP-based immunotherapy. PC formation has complex effects on immunotherapy since it can diminish (“immune blinding”) or enhance the immune response in an uncontrolled manner (“immune reactivity”). Here, future perspectives of the field including the latest advances towards the use of personalized protein corona in cancer immunotherapy are also discussed.

## 1. Introduction

Immunotherapy has attracted special attention in recent years as a novel cancer therapy, achieving durable and long-term responses in patients through the use of monoclonal antibodies or adoptive cell therapy [1,2]. However, immunotherapeutic resistance undermines the efficacy of these types of treatments [3]. Cancer vaccines based on antigen-specific immune responses against tumor-associated antigens present potential advantages in combination with other immune therapeutics [4,5].

Nanoparticles (NPs) are ideal candidates as platforms for developing novel cancer nano-vaccines due to their excellent physical and chemical properties, good colloidal stability, low toxicity, good biocompatibility, and due to the possibility of simultaneously loading adjuvants and antigens [6,7]. Despite these advantages, NPs frequently present colloidal stability problems under physiological conditions, poor circulation time in the bloodstream, and unwanted interactions with cells of the reticuloendothelial system such as macrophages [8]. However, one of the biggest limitations for nanoparticle-based therapies is the formation of the protein corona (PC) on the nanoparticle surface [9,10]. PC can change the physicochemical properties of the nano-formulations, and hence the desired therapeutic effect. This issue is especially important in the case of immunotherapy, where the recognition of the molecules on the surface of NPs is essential to trigger an immune response. In this review, we will summarize the latest research on PC and its effects on the immune-related treatment modulation.

The immune system is composed of different immune-organs and immune cells, which in communication with other non-immunological organs and cells are able to protect humans against foreign bodies or microorganisms. This protection is performed in parallel with the maintenance of tolerance towards self-antigens, contrary to for example in autoimmunity diseases or allergies in which this tolerance gets compromised [11,12,13]. The immune system generates two types of immune responses: the innate and the adaptive immune response. The first involves dendritic cells (DCs), natural killers (NKs), and macrophages, among other cells, and it is responsible for the very first barrier that pathogens meet. DCs and macrophages reside in many different tissues and when a foreign body is recognized by the pattern recognition receptors (PRRs), they trigger an inflammation response which serves as an alert to other immune cells (innate and adaptive cells). The innate immune cells that migrate to the inflammation area enhance the alert signal, and the adaptive immune cells (lymphocytes) start a specific response against the foreign body. The main differences between the two immune responses are the response time and the specificity, the latter being a unique feature of the adaptive immune response [14,15]. Sometimes, the immune system itself can cause pathologies by losing the control over the small auto-reactive population of cells normally present in the body, overreacting against the body itself, and leading to autoimmune diseases [16]. Another disorder, which is not considered a disease, is allergy. In the case of allergy, the presence of an antigen that should not be immunogenic provokes a strong immune response [17]. What is more, immune disorders can be also caused by a clever pathogen such as human immunodeficiency virus (HIV), that misleads the immune system and provokes an immunodeficiency [18].

**Figure 1:**
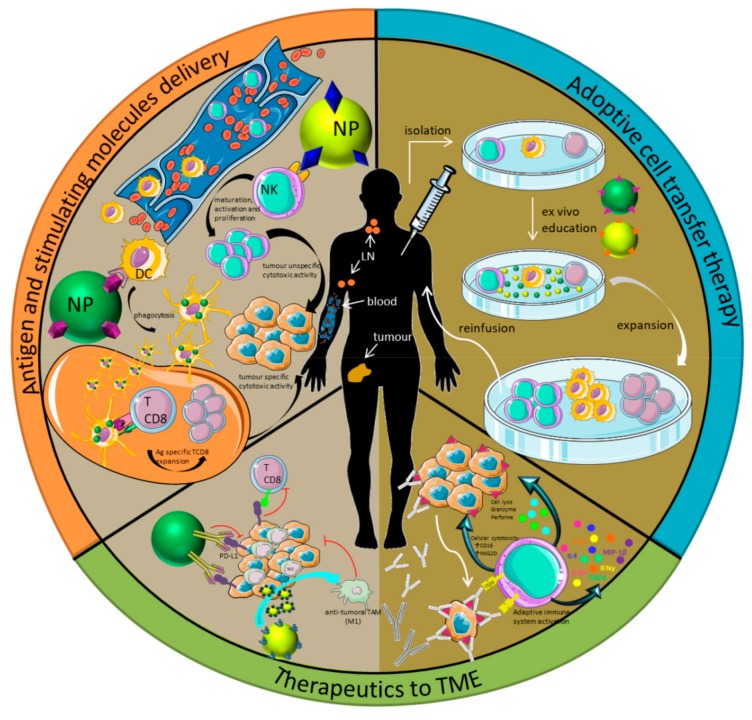
Classification of immunotherapy strategies. Active (pale brown on the left) vs. passive immunotherapy (dark brown on the right). The three main immunotherapy strategies used in cancer treatment are shown in the figure separately: (1) Antigen and stimulating molecule delivery (orange); (2) Adoptive transfer therapy (blue); and (3) Therapeutics to the tumor microenvironment (TME) (green). Figure adapted from [20,27]. NP: nanoparticle; NK: natural killer; DC: dendritic cell.

Immunotherapy focuses on exploiting the immune system of the patient to treat a disease. Immunotherapy can be applied for two main purposes, inhibition or enhancement of the immune system, depending on the desired effect. In terms of therapy, the most general classification is the division between active or passive immunotherapy (Figure 1) [19]. This classification depends on how the therapy stimulates the immune system. Active immunotherapy includes treatments aimed at priming the immune response against antigens. In this category are included vaccines and checkpoint inhibitors for example, since they are only immune active with the collaboration of the host immune system. In contrast, passive immunotherapy involves an intrinsic immune response, mediated by the administration of immune stimulating molecules, like cytokines or antibodies. This is the case of adoptive transfer therapy and monoclonal therapeutic antibodies (mAb), among others [20].

Although cancer is not considered an immune disease, the pathology emerges from failures in the immune response. In a normal situation, cancer cells should be eliminated from the body through the immune response. However, in cancer pathology, these aberrant cells (cancer cells) confuse the immune system and cause immune-resistance by promoting immunosuppressive signals. This immune escape results in the tumor progression [21,22]. Considering this effect, treating cancer in combination with immune-stimulating therapies arises as a promising therapeutic strategy. This strategy was applied for first time in the late state of nineteenth-century when William B. Coley treated a patient who had an inoperable sarcoma and *Streptococcus* sp. He observed how the tumor volume decreased as a consequence of the immune response boosted by the bacterial infection [23]. During the last two decades, immunotherapies have been improved, not only as proof of concept at the pre-clinical stage but also in clinical research, which has led to ongoing clinical trials (NCT01898793, NCT03068819, and NCT02782546) [24]. In depth, several new treatments have been approved in the last decade (Table 1) and James P. Allison and Tasuku Honjo were awarded the Nobel Prize in Physiology or Medicine 2018 for their work in the field of cancer immunotherapy and the discovery of cancer therapy by inhibition of negative immune regulation [25,26].

In addition to the general classification of immunotherapies in active or passive therapies, there are three main strategies in which immunotherapies could be grouped (Figure 1), at least in cancer immunotherapy. The first strategy is the use of nanoparticles (NPs) as delivery systems for antigens or stimulating molecules (Figure 1, orange section). NPs could act as nanocarriers for stimulating molecules, which will be transported to target specific receptors and trigger the immune system activation or inhibition. Moreover, if the transported molecule is an antigen they are known as nano-vaccines. In this case, the objective of the strategy is to reach the lymph nodes (LNs) to target the T lymphocytes and generate a specific cytotoxic response against the tumor [47,48]. The second strategy is the adoptive cell transfer therapy (Figure 1, blue section), in which the immune cells are isolated from the patient, educated ex vivo [49,50,51], and reinfused into the patient. Finally, the third strategy is the delivery of therapeutics to the tumor microenvironment, by the direct infusion of the therapeutic agents into the tumor area (Figure 1, green section). One example is the use of checkpoint inhibitors that recognize programmed death-ligand 1 (PD-L1) or cytotoxic T lymphocyte antigen 4 (CTLA-4) ligands on the cell membrane of tumor cells and block the inhibition mediated by those checkpoint inhibitors that prevent T cells from killing cancer cells [52,53]. Other promising strategies are based on the polarization of the tumor-associated macrophages (TAMs) from M2 to M1, which present anti-tumor properties [29,54]. These strategies present some limitations including (1) the determination of the more effective small molecules in TAM polarization, (2) the difficulty in preferentially delivering small molecules to TAMs in vivo, and (3) reaching sub-optimal therapeutic efficacy. Rodell et al. described the polarization of TAMs from M2 to M1 in multiple tumor models in mice [55].Using a monotherapy based on an agonist of the toll-like receptors (TLRs)loaded in β-cyclodextrin nanoparticles they altered the functional orientation of the tumor immune microenvironment towards an M1 phenotype, leading to a controlled tumor growth and the protection of the animals against tumor re-challenge. In addition, using this nano-formulation in combination with the immune checkpoint inhibitor anti-PD-1, they also observed improved immunotherapy response rates in a tumor model resistant to anti-PD-1 therapy [55]. More recently, this strategy in combination with CD47 antagonists has been considered to inhibit cancer recurrence and metastasis effectively. Chen et al. have developed an in situ formed immunotherapeutic bioresponsive gel that controls both local tumor recurrence after surgery and development of distant tumors. In this work, calcium carbonate nanoparticles pre-loaded with the anti-CD47 antibody were encapsulated in a fibrin gel and scavenge H^+^ in the surgical wound, allowing polarization of tumor-associated macrophages to the M1-like phenotype. The released anti-CD47 antibody blocks the ‘don’t eat me’ signal in cancer cells, thereby increasing phagocytosis of cancer cells by macrophages. In this way, macrophages can promote effective antigen presentation and initiate T cell mediated immune responses that control tumor growth [37].

Over the last years, immunotherapy has become the main promise in cancer treatment. However, there are still some challenges related to the successful use of immunotherapy in cancer. One of these limitations is primary or acquired resistance. Cancer cells may change the expression levels of their membrane proteins, for example, the programmed death-ligand 1 (PD-L1). The binding of the PD-L1 to its receptor PD-1 (present in the surface of immune cells) involves the delivery of inhibitory signals that function as a brake for immune responses. Therefore, through the over-expression of PD-L1, cancer cells send immune inhibitory signals through PD-L1/PD-1 complex favoring immune escape and accelerating tumor progression. This is the mechanism by which cancer tissues limit the host immune response via up-regulation of the abovementioned PD-L1 and its binding to the programmed death-1 (PD-1) receptor on antigen-specific CD8+ T cells (termed adaptive immune resistance) [56,57]. Therapies targeting these inhibitory receptors, for example PD-1 receptor, have shown outstanding rates of durable clinical responses in patients with different cancer types [58]. Another limitation of cancer immunotherapy is the difficulty of reaching the tumor location away from the injection route. To overcome this limitation, the treatment dose is generally increased to achieve an efficient drug dose in the tumor [59]. However, this dose increase could trigger undesired side effects harmful to the patients. This limitation makes NPs ideal candidates for the delivery of cancer immunotherapy since they follow different pharmacokinetics and pharmacodynamics compared to free drugs [60,61].

Over the last two decades, NPs have been widely explored for their use in biomedical applications. Nevertheless, immunotherapy was not the first biomedical use of NPs. A few years ago, editors from *Science* named immunotherapy as the “Breakthrough of the year” [1]. After this fact, NPs started being used as immunotherapeutic agents in addition to chemotherapeutic agents. Moreover, some anticancer treatments based on NP formulations have been approved by the FDA, and are currently in clinical trials (Table 1). NPs have shown great promise in the treatment of cancer as cancer nano-immunotherapies. However, these systems face issues concerning stability in physiological media, PC formation, and accumulation in the target tissue. Among several important issues, the formation of a PC around the NPs in the presence of biological fluids plays an important role, mainly in changing the physicochemical properties of the nano-formulations, with consequent diminished in the therapeutic efficacy of nanomedicines. Furthermore, the modification of the surface of the particles is patient-specific and the formation of a PC may have additional undesired effects on the performance of the NPs including loss of efficacy of targeting moieties, undesired flagging by the complement, unspecific uptake by immune cells, and immunotoxicity.

## 2. Formation of Protein Corona: Effect on NP-Based Immunotherapy

In the last years NPs have been widely explored for their use in biomedical applications. In this context, it is important to understand the interactions occurring at the interface between NPs and biological fluids to predict the fate of injected NPs. It is commonly accepted that the interaction of the NPs and biological fluids is a consequence of several factors.NP size, shape, charge, or coating agents are critical [62,63,64,65,66,67,68,69], but the characteristics of the biological fluids are also very important (ionic strength, protein concentration, pH, and temperature) [70]. Once NPs are exposed to biological fluids, they interact with active biomolecules (mostly proteins, but also sugars, nucleic acids, and lipids) and PC is formed around them by the unspecific absorption of proteins on the surface of the NPs. This effect gives the NPs, upon protein corona formation, a different biological identity compared to bare NPs. The physicochemical properties of the bare NPs such as size, surface charge, surface composition, and functionality, change due to the PC formation. Therefore, the characterization of the properties of NPs after their exposure to a biological fluid has become mandatory for two purposes, to understand how these new characteristics affect the behavior of the nano-formulation in vivo and to design strategies to avoid the PC formation. In this context, Zhou et al. disclosed that the dynamic structure of nanoparticle surfaces can affect the protein adsorption kinetics and thus the interaction between nanoparticles/adsorbed proteins and cells [71].

Recently, the scientific community has been moving from the mere evaluation of the impact of the PC on the physicochemical properties of NPs to the evaluation of the impact on their behavior in physiological systems. Furthermore, a large number of studies have provided much insight into the layer thickness and composition of the PC, and the adsorption kinetics under different experimental setups. Many techniques have been used to measure the absorption of proteins around the NPs such as UV-visible spectroscopy (UV/Vis), dynamic light scattering (DLS), transmission electron microscopy (TEM), and fluorescence correlation spectroscopy (FCS) [72,73,74]. Another non-optical method that allows for the measurement of PC formation in complex media such as blood is ^19^F diffusion measured by nuclear magnetic resonance (NMR) [75]. In addition, different techniques such as surface plasmon resonance (SPR), isothermal titration calorimetry (ITC), differential centrifugal sedimentation (DCS), and quartz crystal microbalance (QCM) have been used to quantify the affinities of proteins for NPs [76,77,78,79,80]. Nevertheless, liquid chromatography–mass spectrometry (LC-MS) is probably the most powerful tool to identify proteins present in the protein corona [81,82].

**Figure 2:**
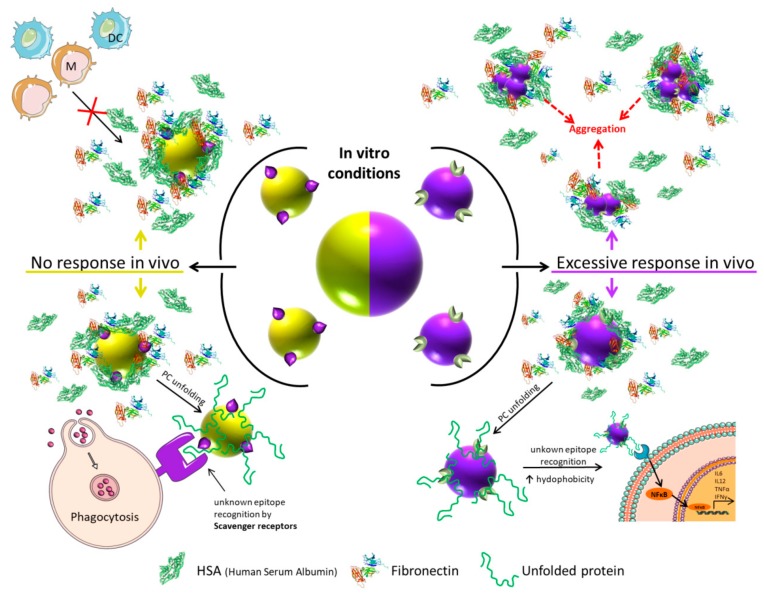
Nanoparticle-based immunotherapy failure because of protein corona (PC) formation. No-response (**left**) vs. excessive or uncontrolled response (**right**). Top left panel: Immune cells are not able to recognize the molecules on the surface of the NPs because the PC covers the NPs partially or totally. Bottom left: NP phagocytosis by macrophages because of the denaturalization of the proteins (in green) on the surface of the NPs. Top right: Aggregation of NPs triggers toxic effects by strange-body recognition by immune system. Bottom right: Nuclear Factor κB (NF-κB) translocation to the nucleus because of the recognition of denatured proteins on the surface of the NPs.

PC formation is especially relevant in the field of the immunotherapy, since in order to trigger an immune response it is essential the interaction between antigens or other molecules and their receptors. In this sense, has been demonstrated that the PC can have a dual role in biomolecular recognition (Figure 2). In some cases, the PC hides the antigen/molecule on the NPs surface, thereby inhibiting the interaction with its specific receptor (this immune-escape process could be defined as “*immune-blinding*”); and in other cases the PC contains proteins that act as ligands for receptors on specific immune cells and triggers undesirable immune responses (immune reactivity) [83,84]. Additionally, PC can provoke the phagocytosis by monocytes and macrophages [83] and in consequence could avoid the recognition of the stimulating molecules exposed in the surface of the NPs.

### 2.1. Immune-Blinding as a Consequence of PC Formation

As shown in Figure 2, the immune-blinding could be promoted by two main mechanisms. On the one hand, PC may fully or partially covers the antigens/molecules present on the surface of the NPs, and in consequence, the specific stimulation will be low or fail and consequently the immune response will not occur. Shanehsazzadeh et al. described a good example on how PC can induce immune-blinding on a nano-formulation in vivo. The uptake of NPs functionalized with anti-mucin1 protein (anti-MUC-1) antibody was nine times higher in MUC-1-positive cells than in the MUC-1 negative cells in vitro. However, in the in vivo mice model, the antibody-functionalized NPs showed higher distribution in blood and muscle than in tumor. The conclusion of this work was that the PC covered the targeting molecules and in consequence, the tumor uptake in vivo was reduced [85]. Conversely, other NP formulations based on the virus-like particle (VLP)-functionalized anti-MUC-1 antibody displayed higher tumor accumulation and also higher metastatic protection in mice than non-functionalized NPs [86]. These differences in vivo evidence the importance of the NP composition in the possible immune-blinding effect promoted by the PC, as was also shown in a study in which a differential PC formation was observed depending on the PEGylation grade of NPs [87]. In the case of nano-vaccine based therapies, the PC also plays a critical role in the uptake of the nano-formulation by dendritic cells, which is a critical step for an effective therapeutic response [62,88].

**Figure 3:**
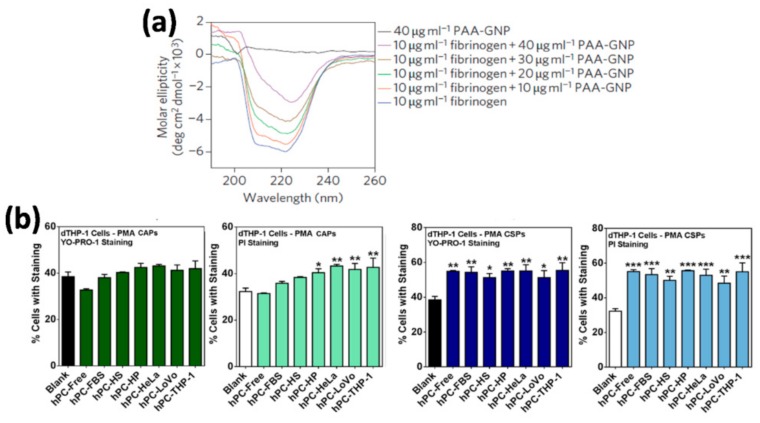
(**a**) Protein denaturalization on the surface of NPs. Secondary structure of the fibrinogen protein measured by circular dichroism after being incubated with different concentrations of 5-nm poly(acrylic acid)-coated gold nanoparticles (PAA–GNPs).Figure from [94]. (**b**) Effect of protein corona formation and composition on early apoptosis and cell death responses in THP-1 cells, a human Leukemic monocytes cell line. Poly (metacrylic acid) hollow particles (PMA CAPs) (in green) and PMA cores-shell particles (PMA CSPs) (in blue) were exposed to various environments (different media, serum free control, fetal bovine serum (FBS), human serum (HS), human plasma (HP), and culture media with three different cell lines HeLa, LoVo, and THP-1) to acquire a hard protein corona (hPC). The early apoptosis (YO-PRO-1 staining) and cell death (PI staining) were measured by flow cytometry. Data are shown as the mean ± standard error of at least four independent experiments, with at least 10,000 cells analyzed in each experiment. * *p*< 0.05, ** *p*< 0.01, *** *p*< 0.001, versus the column of “Blank” (indicating untreated cells) (one-way ANOVA Dunnett’s multiple comparison test). Figure from [95].

On the other hand, the blinding effect can be due to the homeostatic function of immune cells. Macrophages have so-called scavenger receptors that recognize biological patterns on strange bodies [89]. Sometimes, the structure of the proteins that formed the PC is altered during the PC formation on the surface of the NPs [90], and in consequence usually unexposed epitopes are presented to the immune system (Figure 3a). Macrophages could recognize these epitopes and phagocyte the NP–PC complexes through the scavenger receptors (Figure 2) [91,92]. This situation could be solved changing the physicochemical properties of the NPs and in consequence reducing the uptake of the NPs by macrophages [93].

### 2.2. Immune-Response or Immune-Reactivity as a Consequence of PC Formation

The difference between a controlled and an uncontrolled immune response, which could be called immune-reactivity, is not always clear. As immunotherapy enhances the very complex immune system, this stimulation has to be carefully designed. In nano-immunotherapy it is essential to consider that the contact of NPs with a biological fluid will most probably provoke the formation of a protein corona on the NPs surface that could trigger an excessive immune activity, resulting mostly in inflammation [93]. This phenomenon is commonly related to the excessive production of the pro-inflammatory cytokines such as TNFα, INFγ, IL-6, and IL-12 and the exacerbated inflammatory response associated to high levels of these cytokines that could destroy healthy cells and tissues. In this context, Dai et al. observed differences in pro-inflammatory cytokine secretion and immune cell apoptosis when studying the interaction of NP–PC complexes, formed in various biologically relevant environments, with macrophages. They observed that the NP–PC complexes either increased or mitigated the secretion of a specific cytokine, depending on the environment where the protein corona was formed (Figure 3b) [95]. On the other hand, although it has been demonstrated that the PC could trigger pro-inflammatory responses and cell death, some works suggest that PC could also have protective properties. Escamilla-Rivera et al. studied the role of the PC as potential protector for Reactive Oxygen Species (ROS)-induced cytotoxicity and pro-inflammatory response in macrophages exposed to iron oxide NPs. They observed that the reduction in Iron Oxide Nano-Particle (IONP) cytotoxicity can be attributed to the PC shielding against ROS generation and pro-inflammatory response in macrophages [96].

The formation of protein corona on the NP surface does not always trigger an excessive immune activity per se, as has been previously described. Sometimes, the excessive immune activity could be associated with the presence of unfolded proteins in the NP–PC complexes. In the same way that macrophages can recognize some unfolded proteins present in NP–PC complexes through the scavenger receptors and phagocyte them, other specific receptors can recognize unfolded proteins present in NP–PC complexes and trigger an exacerbate inflammation response. Deng et al. described that negatively charged poly(acrylic acid) (PLA)-conjugated gold NPs bind to and induce unfolding of fibrinogen, which promotes interaction with the integrin receptor, Mac-The activation of Mac-1 receptor increases the NF-κB signaling pathway, resulting in the release of inflammatory cytokines [94]. However, not all NPs that bind to fibrinogen showed this effect, which illustrates the influence of the physicochemical properties of the NPs on the cellular innate immune response. The role of the physicochemical properties of the materials, such us surface chemistry and wettability, on the formation of PC in human serum and the subsequent effects on the cellular innate immune response have been also investigated. Visalakshan et al. demonstrated that the amount and identity of proteins adsorbed on the surface of the different materials were strongly influenced by surface chemistry and wettability, which led to a distinct immune response from macrophages. Hydrophilic surfaces mostly adsorbed dysopsonin and albumin, which induced a greater expression of anti-inflammatory cytokines by macrophages. In contrast, hydrophobic surfaces mostly adsorbed Immunoglobulin G2 (IgG2) type opsonin, which caused increased production of pro-inflammatory signaling molecules [97]. Therefore the identity of the adsorbed proteins on the surface of the NPs has also an important role in triggering excessive immune responses. The administration of any nanomaterial to animals or humans results in the adsorption of proteins onto the nanomaterial surface and the subsequent complement activation. The complement pathway activation is translated into an exacerbate inflammation response. This effect may be related to the observations that the presence in the PC of the third component of the complement protein (C3) affects the immune cell recognition of nanomedicines [98,99,100]. To avoid the nanomaterial-induced complement activation, many researchers have used highly biocompatible materials such as zwitterionic polymers as well as hydrophilic nanoparticles which decrease the protein adsorption [101], and biomaterials already wrapped with “self” proteins such as CD200 [102]. More recently, cell membrane coatings have emerged as a new class of coatings that enable the camouflage of NPs for evading immune clearance and lessen the complement activation by nanoparticles [10,103,104]. For example, Fan et al. developed a coating based on red blood cell (RBC) membranes that was able to camouflage the particles from the immune system and significantly reduced the number of infiltrating neutrophils [105].

These works and other many studies map out relationships between the physicochemical properties of the NPs and other materials, the protein corona formation, and subsequent cellular innate immune responses [106]. The potential outcomes of these studies can guide the development of new nanomaterials to modulate serum protein adsorption and to avoid undesirable innate immune responses.

## 3. Future Perspectives

Nanoparticles have shown great promise in the treatment of cancer and as synthetic nano-vaccines. However, these systems face issues concerning stability under physiological conditions, protein corona formation, and accumulation in the target tissue. One of the biggest limitations for nanoparticle-based therapies is the formation of a protein corona (PC) on the nanoparticle surface and its effect on their biological performance. Therefore, it is fundamental to include systematic studies on NP–PC complexes and to define the biological identity of the final nano-formulation in the field of nano-therapy [107,108]. These studies will be useful in order to predict the physicochemical changes, such as protein absorption, of the NPs that could affect NP-based immunotherapies causing “immune blinding” or immune reactivity.

**Figure 4:**
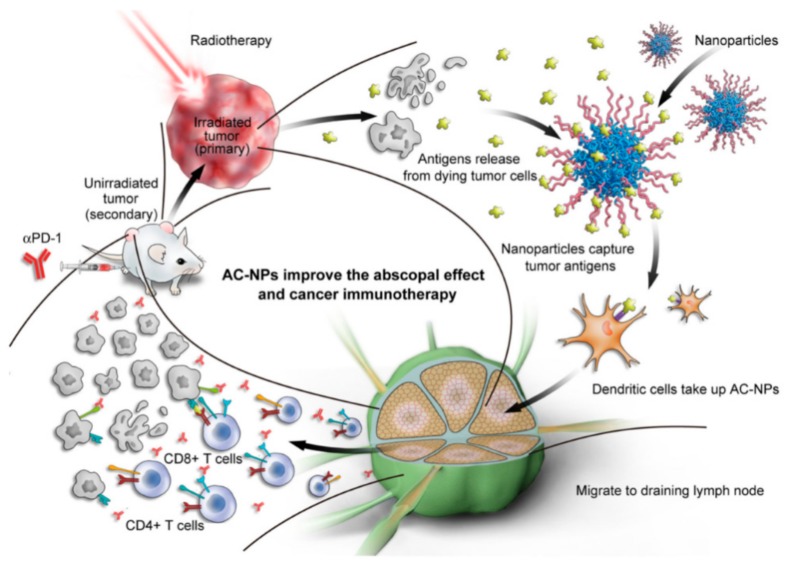
The protein corona in cancer immunotherapy. Schematic description of an NP–PPC based therapy mechanism in which radiotherapy induces the release of tumor-derived protein antigens (TDPAs) that are integrated in the PC and promote the antigen presentation and the anti-cancer immune activation. This approach is applied in combination with checkpoint inhibitor therapy. Figure from [117]. PPC: personalized protein corona. AC-NPs refers to Antigen Capture-Nano Particles.

Even though protein adsorption and PC formation appear to be two of the biggest limitations for nanoparticle-based therapies, there is a possibility to revert this situation to a positive scenario. This is the case of the personalized protein corona (PPC) [109,110,111]. Recently, it has been shown that the protein corona is strongly affected for example by specific diseases in patients from which the plasma is obtained. Therefore, the same nanomaterial incubated with plasma proteins from patients with different pathologies generates protein coronas with different compositions, giving rise to the concept of personalized protein corona [109]. In recent years, the PPC has become a very interesting approach to screen protein biomarkers in plasma for early diagnosis and treatment of different diseases [102,112]. Specially, the PPC could be revolutionary in the field of cancer immunotherapy. In this sense, one of the best examples of NP–PPC based cancer immunotherapy was designed taking into account a key clinical approach to improve cancer immunotherapy: the combination of radiotherapy with checkpoint inhibitors. This combinatory therapy produces an immune stimulation by radiation-induced pro-inflammatory protein production and increases the exposure of immune cells to cancer-specific antigens that are released following radiotherapy-induced cancer cell death [113,114,115,116]. Therefore, NPs could be used to capture tumor-derived protein antigens (TDPAs) released during radiotherapy and transporting them to antigen-presenting cells (APCs), thereby promoting anti-cancer immunity (Figure 4) [117].

In addition to the PPC, PC could also be modified to modulate the NP–PC biological properties. In this context, Wan et al. demonstrated that the post-modification (de-glycosylation) of the NP–PC complexes had a critical role in the improvement of NP-cell interaction and in the cellular uptake. The de-glycosylation of the NP–PC complexes enhanced cell membrane adhesion to two types of THP-1 differentiated macrophages (polarized to M1 and M2), leading to an increase in NPs uptake [118].

Within the context of the recent developments in cancer immunotherapy research and in the impact of protein corona formation on the efficacy of nanotherapies, a few aspects can be highlighted to establish the path for the future developments. The complexity of the effect of protein corona formation in nanomedicine and in particular in the context of nano-immunotherapy reveals the need of detailed fundamental studies on the protein corona formation and the interaction of protein corona with the immune system. Recently, big research efforts have been made toward developing “stealth” nanomaterials to avoid the formation of the protein corona. However, these approaches had shown limited success, and there is still room for future surface engineering advances to minimize the formation of the protein corona.

An alternative ground-breaking path is the use of the protein corona to endow biological functions to the nanoparticles by fine tuning and modifying the protein corona composition. The encouraging preliminary studies on the use of personalized protein corona in cancer immunotherapy described above establish the basis for this research direction. Therefore, the next frontier in nano-immunotherapy is the design of advanced nanomaterials with the personalized protein corona, in which the previous drawbacks related to the formation of the protein corona are turned into advantages. This approach is framed in the broader context of the recent boost of the personalized medicine.

## Figures and Tables

**Table 1:** Recently studied nanoformulations for immunotherapy. Some nano-formulations are approved by Food and Drug Administration (FDA) and others are in clinical trials [28].

	Compound Name	Formulation Description	Chemotherapy	Immunotherapy Type	Route of Immunization	Clinical Trials	Approved by the FDA	Ref.
Immunotherapy	Ferumoxytol (Ferahem^®^)	IONP	No	Active	TME→ M2-like macrophages to M1-like		Yes, for anemia and kidney diseases	[29]
	eCPMV	VLP of cowpea mosaic virus	No	Active	Neutrophil activation in the TME	-	-	[30]
	RNA-LPX (Lipoplex^®^)	RNA-loaded liposomes	No	Active	DC maturation, Tcell response, inflammatory response	Phase I (2016)		[31]
	PTX-LDE	Paclitaxel-loaded lipid core NPs	Yes	Active	DC maturation [32]	Phase II (2017)		[33]
	MRX34	miRNA-34a-loaded liposome	No	Passive	Downregulation of immune evasion tumor genes	Phase I (2016)		[34]
	nab-Paclitaxel (Abraxane^®^)	Paclitaxel-loaded albumin NPs	Yes	Not applied right now	DC maturation	Phase III (2017)	Yes, for cancer treatment	[35,36]
	aCD47@CaCO_3_	Anti CD47-loaded CaCO3 NP in fibrinogen solution	No	Active	After surgery, and with the addition of thrombin, aCD47@CaCO3 forms a immunotherapeutic gel in situ in the TME	-	-	[37]
	Sipuleucel-T (Provenge^®^)	ex vivo DCs	No	Active	Vaccine		Yes, for prostate cancer	[38]
	Blinatumomab (Blincyto^®^)	Bi-specific T cell engager (BiTE). Specific to CD19 and CD3.	No	Passive	BiTE targeting CD19 (malignant B cell) and CD3 (T cell) and cytotoxicity effect against B cells.		Yes, for Philadelphia negative Acute lymphocytic leukaemia (ALL)	[39]
	Talimogene Laherparepvec (T-VEC)	Injectable modified herpes virus	No	Active	Vaccine		Yes, for advanced melanoma	[40]
No immunotherapy	BIND-014	Docetaxel-loaded Poly-Lactic Acid (PLA) NP and Prostate-Specific Membrane Antigen (PSMA) in the surface	Yes	No	-	Phase II(2018)	No	[41,42]
	SPIO	Super paramagnetic iron oxide NPs	No. Only for imaging		-	-	Yes for imaging	[43]
	Doxil^®^	Dox-loaded liposome	Yes	No	-	-	Yes	[44]
	Marqibo	Vincristine-loaded liposome	Yes	No	-	-	Yes, for Ph negative ALL	[45]
	Ontak^®^	Protein NPs	Yes	No	-	-	Yes, for cutaneous T cell lymphoma	[46]
